# Brief Video-Delivered Interventions to Reduce Anxiety in Older Veterans: A Pilot RCT

**DOI:** 10.1093/geroni/igab046.2178

**Published:** 2021-12-17

**Authors:** Christine Gould, Chalise Carlson, Lauren Anker, Ruth O'Hara, Julie Wetherell, Mary Goldstein, Sherry Beaudreau

**Affiliations:** 1 VA Palo Alto Health Care System, Palo Alto, California, United States; 2 Stanford University School of Medicine, Palo Alto, California, United States; 3 VA San Diego / UCSD, La Jolla Village, California, United States; 4 VA Central Office, Palo Alto, California, United States; 5 VA Palo Alto / Stanford University School of Medicine, VA Palo Alto, California, United States

## Abstract

Older Veterans with anxiety disorders encounter barriers to receiving mental health services that may be overcome by using brief technology-delivered interventions. To address this, we conducted a pilot randomized controlled trial (RCT) comparing the effects of a guided self-management intervention called BREATHE, a 4-week video-delivered (DVD/internet) intervention and a psychoeducation control (Healthy Living; HL) on anxiety symptom severity. Older Veterans with anxiety disorders (N = 48; 87.5% men; Mean age = 71.77 ± 6.2 years) were randomized to BREATHE or HL. Regarding intervention delivery modality, 67% used DVDs, 23% used the internet, 4% used both to access their assigned intervention. Both groups experienced significant declines in affective anxiety from baseline to 8 weeks followed by an increase in symptoms (i.e., quadratic pattern). HL had significant declines in somatic anxiety, whereas BREATHE did not experience such declines. The longitudinal effects and Veteran satisfaction will be further described in the presentation.

